# Molecular characterization of a Chinese family carrying a novel C4329A mutation in mitochondrial tRNA^Ile^ and tRNA^Gln^ genes

**DOI:** 10.1186/1471-2350-15-84

**Published:** 2014-07-23

**Authors:** Yuqi Liu, Yang Li, Jinliao Gao, Chao Zhu, Yunfeng Lan, Jie Yang, Zongbin Li, Minxin Guan, Yundai Chen

**Affiliations:** 1 The Institute of Geriatric Cardiology, Cardiac Department, Chinese PLA General Hospital, Beijing, China; 2 Attardi Institute of Mitochondrial Biomedicine and Zhejiang Provincial Key Laboratory of Medical Genetics, Wenzhou Medical College, Wenzhou, Zhejiang, China; 3 Division of Human Genetics, Cincinnati Children’s Hospital Medical Center, Cincinnati, OH, USA; 4 Department of Genetics, College of Life Sciences, Zhejiang University, Zhejiang, China

**Keywords:** Mitochondria, Hypertension, Mutation, tRNA

## Abstract

**Background:**

Hypertension is a very common cardiovascular disease influenced by multiple genetic and environmental factors. More recently, there are some studies showed that mutations in mitochondrial DNA have been involved in its pathogenesis. In this study we did further investigations on this relationship.

**Methods:**

Epidemiological research found a Han Chinese family with probable maternally transmitted hypertension. Sequence analysis of the whole mitochondrial DNA was detected from all the family members. And evaluations of the clinical, genetic and molecular characterization were also performed.

**Results:**

Matrilineal relatives within the family exhibited varying degrees of hypertension with an onset age of 48–55 years. Sequence analysis of this pedigree showed a novel homoplasmic 4329C > G mutation located at the 3’ end of the tRNA^Ile^ and tRNA^Gln^ genes that was absent from 366 Chinese controls. The cytosine (C) at 4329 position was very important in the structural formation and stabilization of functional tRNAs, which was highly conserved in mitochondria of various organisms and also contributed to the high fidelity of the acceptor arm. Cells carrying this mutation were also shown to harbor mitochondrial dysfunctions.

**Conclusions:**

The C4329G point mutation in tRNA^Ile^ and tRNA^Gln^ was involved in the pathogenesis of hypertension, perhaps in association with other modifying factors.

## Background

Essential hypertension (EH) is one of the most important modifiable risk factors for cardiovascular disease (CVD) and renal disease in worldwide. In China, there is a prevalence estimate of 27.9%, or 133 million, hypertensive adults [1]. Hypertension is also a major risk factor for the development of CVD and renal morbid- and fatal events [2].

The etiology of hypertension is not well understood because it can be caused by single or multiple genetics, and also associated with environmental and other individual factors. Of the genetic factors, maternal transmission of hypertension has been implicated in some pedigrees, suggesting that mutations in mitochondrial DNA (mtDNA) may contribute to the pathogenesis of EH [3-7]. More recently, several mtDNA point mutations have been proved in association with hypertension. These mutations include T3308C in *ND1*[8], A4435G in tRNA^Met^[9], A4295G and A4263G in tRNA^Ile^[10,11], and A4401G in tRNA^Met^ and tRNA^Gln^[12].

To identify novel mtDNA mutations involved in the pathogenesis of hypertension in Chinese population, we initiated a systematic and extended mutational screening of mtDNA in a large cohort of hypertension subjects from the Geriatric Cardiology Clinic at the Chinese PLA General Hospital. Here, we reported a novel point mutation C4329G in tRNA^Ile^ and tRNA^Gln^ genes carried by a Chinese Han family with maternally inherited hypertension.

## Methods

### Subjects

**Figure 1:**
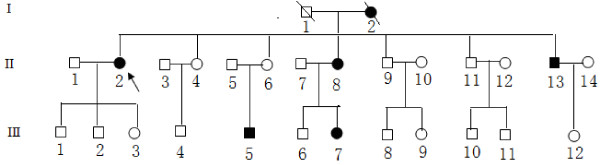
**Pedigree chart of the Chinese family with hypertension.** Affected individuals are indicated by filled symbols. Arrowhead denotes proband.

As part of a genetic screening program for hypertension, a Chinese Han family with maternal inherited hypertension was found at the Institute of Geriatric Cardiology at the Chinese PLA General Hospital. An experienced physician measured the systolic and diastolic blood pressures of subjects with a mercury column sphygmomanometer using a standard protocol. The first and the fifth Korotkoff sounds were taken to be indicative of systolic and diastolic blood pressures, respectively. The average of three such readings was taken as the examination blood pressure. Hypertension was defined according to the recommendation of the Joint National Committee on Detection, Evaluation and Treatment of High Blood Pressure (JNC VI) [13] as a systolic blood pressure of 140 mmHg or higher and /or a diastolic blood pressure of 90 mmHg or greater. All the members of this family underwent a thorough physical examination, a laboratory assessment of CVD risk factors, and routine electrocardiography (Figure 1). All subjects were evaluated to identify both personal and medical histories of hypertension and other clinical abnormalities. DNA samples of the family and 366 healthy controls were aquired for sequence analysis. Informed consents were obtained from all participating members, under protocols approved by the ethics committee of the Chinese PLA General Hospital.

### mtDNA sequencing and analysis

Genomic DNA was isolated from whole blood cells of participants using Puregene DNA Isolation Kits (Gentra Systems, Minneapolis, MN). The entire mitochondrial genome of all the participants were PCR-amplified in 24 overlapping fragments using sets of light-strand and heavy-strand oligonucleotide primers [14]. Each fragment was purified and subsequently analyzed by direct sequencing in an ABI 3700 automated DNA sequencer (Applied Biosystems, Inc., Foster City, CA) using the Big Dye Terminator Cycle sequencing reaction kit. The resultant sequence data were compared with the revised consensus Cambridge sequence (GenBank accession no. NC-012920) [15]. All mtDNA mutations were individually analyzed using the MitoAnalyzer (National Institutes of Standards and Technology, Gaithersburg, MD; http://www.cstl.nist.gov/biotech/strbase/mitoanalyzer.html) and the MITOMAP database [16]. The haplogroups were deduced by comparing the complete mtDNA sequence data with previously reported haplogroup-specific variants [17].

## Results

### Clinical presentation

The proband (II-2) presented with essential hypertension with onset age of 50 years old. Her medical history reported a highest blood pressure reading of 220/120 mm Hg. She first underwent full evaluation at the Chinese PLA General Hospital Geriatric Cardiology Clinic at the age of 60 with a blood pressure 160/80 mm Hg. She suffered from headaches when her blood pressure exceeded 200/100 mmHg, and took oral reserpine and aspirin to prevent stroke when it ranged between 140/80 and 160/100 mmHg. She was diagnosed with diabetes mellitus two years ago for which she was administered oral metformin. Laboratory tests showed a normal range of liver function, normal cholesterol levels, and mild kidney dysfunction (creatinine clearance rate, 81.2 ml/min). Kidney dysfunction was secondary to hypertension. The ECG showed left ventricular hypertrophy. Physical examination revealed no other clinical abnormalities, including hyperlipidemia, vision and hearing impairments, and neurological disorders associated with mitochondrial DNA muation.

The proband’s family located in Shanxi Province in Northern China (shown in Figure 1), the family presented with a maternal inheritance of hypertension. Her mother (I-2) was diagnosed with hypertension at 60 years of age, with a blood pressure of 180/100 mm Hg. She has three brothers and three sisters; one brother (II-13) and one sister (II-8) presented with high blood pressure at the age of 48 years and 55 years, respectively. One of her nieces (III-7) and one of her nephew (III-5) also had hypertension with an onset age of 43 and 40 years old. None of the offspring of the affected father had hypertension. The clinical data of other family members were showed in Table 1.

**Table 1:** Summary of clinical data of the members in the Chinese Pedigree

**Subjects**	**Gender**	**Age of test (yrs)**	**Age of onset (yrs)**	**Systolic pressure (mmHg)**	**Diastolic pressure (mmHg)**	**IVST, mm(6-12 mm)**	**ECG**	**CCr (ml/min)**
II-1	M	63	-	132	60	10	N	89.1
II-2*	F	60	50	160	80	12	LVH	81.2
II-3	M	61	-	128	68	11	N	102.8
II-4	F	58	-	120	60	10	N	103.5
II-5	M	68	-	124	58	9	N	87.9
II-6	F	62	-	130	60	10	N	86.5
II-7	M	70	-	132	70	10	N	92.6
II-8*	F	64	55	150	80	12	LVH	72.3
II-9	M	56	-	128	64	9	N	82.3
II-10	F	52	-	120	66	11	N	87.4
II-11	M	53	-	124	60	10	N	92.7
II-12	F	50	-	132	68	11	N	86.9
II-13*	M	54	48	140	100	11	N	83.2
II-14	F	50	-	128	60	9	N	119.2
III-1	M	40	-	130	60	8	N	122.3
III-2	M	38	-	128	58	8	N	130.2
III-3	F	35	-	118	60	9	N	118.6
III-4	M	33	-	120	58	9	N	108.6
III-5	M	40	40	150	90	12	N	89.1
III-6	M	39	-	124	60	9	N	121.3
III-7	F	44	43	160	90	13	LVH	71.2
III-8	M	30	-	116	60	8	N	114.5
III-9	F	28	-	128	70	9	N	134.8
III-10	M	28	-	126	70	9	N	107.9
III-11	M	25	-	130	70	10	N	126.7
III-12	F	27	-	120	58	9	N	139.6

*These patients received antihypertension treatment. This table shows pretreatment blood pressures. CCr indicates endogenous creatinine clearance; F, female; M, male; IVST, interventricular septal thickness; LVH, ECG showed left ventricular hypertrophy; N, electrocardiography (ECG) was normal.

### Mitochondrial DNA analysis

**Figure 2:**
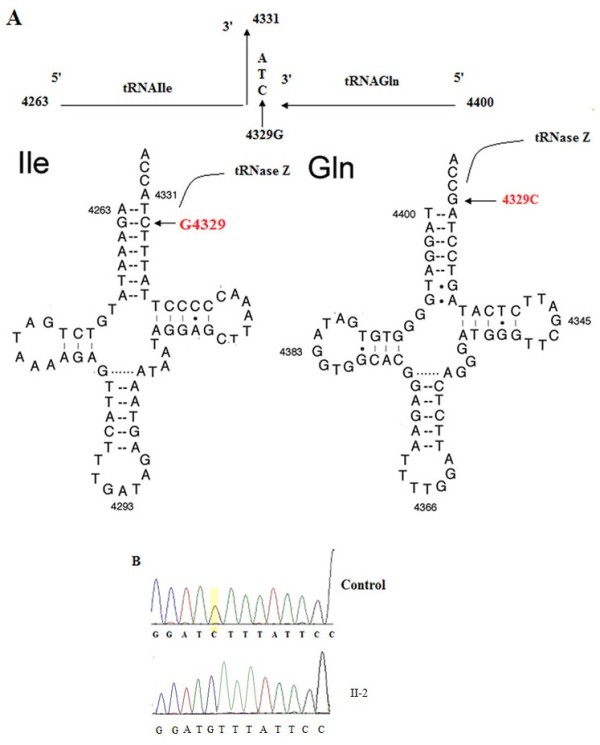
**Identification and qualification of the 4329C > G mutation at the junction between mitochondrial tRNA**^**Ile **^**and tRNA**^**Gln **^**genes. (A)** Schema of the location of 4329C > G in the precursors of tRNA^Ile^ and tRNA^Gln^ genes. Cloverleaf structures of human mitochondrial tRNA^Ile^ and tRNA^Gln^ are derived from Florentz et al. [4]. Processing sites in the mitochondrial tRNA^Ile^ and tRNA^Gln^ precursors were determined for RNase Z. Arrow indicates the position of the 4329C > G mutation. **(B)** Partial sequence chromatograms of tRNA^Ile^ and tRNA^Gln^ genes from an affected individual (II-2) and a married-in control (II-1). Arrow indicates the location of the base changes at position 4329.

Since the transmission pattern of this family presented with maternal inherited hypertension, we supposed that mtDNA maybe involved in the pathogenesis. So we did further analyze on the mitochondrial genome of the family members. We did analyze on the entire mitochondrial gene using PCR amplification and subsequent sequence analysis of PCR fragments derived from proband II-2 and an unrelated Chinese control. As shown in Figure 2B, the point mutation C4329G in tRNA^Ile^ and tRNA^Gln^ genes was identified in the proband. Sequencing of the PCR fragment spanning the tRNA^Ile^ and tRNA^Gln^ genes revealed that this mutation was absent from 366 Chinese controls.

And all the family members had did sequence analysis. All the 14 maternal lineage except the pedigree (I-2), carried the C4329G mutation in tRNA^Ile^ and tRNA^Gln^ genes. The other non-maternal members had no this point mutation. And in these 14 maternal members, there are 5 of them had presented with hypertension. More important, her nieces (III-7) and nephew (III-5) also had hypertension with an onset age of 43 and 40 years old, respectively. And it is very important that none of the offspring of the affected father carried this point muation. We also did segregation analysis on the pedigree, and except for autosomal recessive, autosomal dominant, X-linked patterns. By far the mitochondrial point mutation is the most likely explanation of etiology of hypertension for this pedigree.

**Figure 3:**
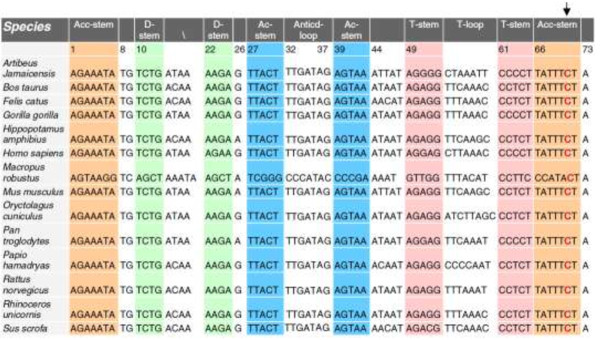
**Alignment of tRNA**^**Ile **^**genes from different species.** Arrow indicates the position of the Acc-stem 71 at the anticodon loop of tRNA, corresponding to the C > G at position 4329.

The C4329G mutation, as shown in Figure 2A, is located at the 3’ end of the acceptor arm of tRNA^Ile^ and tRNA^Gln^[4,18]. Figure 3 presented us that the cytosine residue at this position is highly conserved in all known tRNA^Ile^ and tRNA^Gln^ sequences [4,18]. In fact, nucleoside modifications at this position play a pivotal role in the stabilization of the tertiary structure and the tRNAs’ function [19]. The C4467A mutation in tRNA^Met^ gene which was evolutionary conserved, but was not detected in other maternal members.

To further identify the phenotypic manifestation, DNA fragments spanning the entire mtDNA of the proband II-2 were PCR-amplified. Each fragment was purified and subsequently analyzed by direct sequencing. As shown in Table 2, a comparison of the resultant sequences with the Cambridge consensus sequence and control subject identified a number of nucleoside changes belonging to the Eastern Asian haplogroup H2 [20]. These included eight polymorphisms in the D-loop region, three variants in the 12S rRNA gene, one in the 16S rRNA gene, two in the *ND1* gene, a C4329G mutation in the tRNA^Ile^ and tRNA^Gln^ genes, one variant in the *COII* gene, one in the *COIII* gene, one in each of the ATPase6 and ATPase8 genes, two in the each of the *ND3* and *ND4* genes, and four in each of the *ND6* and *Cytb* genes [21]. Those tRNA, rRNA, and polypeptide variants were further evaluated by phylogenetic analysis of sequences from other organisms such as mouse [22], cow [23] and *Xenopus laevis*[24]. However, none of the polypeptide variants were highly conserved evolutionarily, indicating that they do not have a significant functional consequence.

**Table 2:** **mtDNA variants in 1 Han Chinese subject** (**II**-**2**) **with hypertension and 1 Han Chinese control subject**

**Gene**	**Position**	**Replacement**	Conservation (H/M/B/X)*	**rCRS**^**†**^	**II-2**	**Control**	**Previously reported**^**‡**^	**Amino acid change**
D-loop	73	A to G		A	G	G	Y	
	152	T to C		T	C	C	Y	
	263	A to G		A	G	G	Y	
	310	T to C		T	C	C	Y	
	489	T to C		T	C	C	Y	
12SrRNA	1382	A to C	A/G/G/C	A	C	C	Y	rRNA
	1438	A to G	A/A/C/A	A	G	G	Y	rRNA
16SrRNA	2706	A to G	A/G/G/A	A	G	G	Y	rRNA
	3010	G to A	G/T/G/A	G	A	A	Y	rRNA
ND1	3975	C to T	C/C/A/C	C	T	C	Y	syn
	4260	C to T	C/M/C/T	C	T	T	Y	syn
tRNA Ile	4329	C to G	C/C/C/C	C	G	C	N	
tRNA Met	4467	C to A	C/C/C/C	C	A	C	N	
COII	8020	G to A	G/P/C/A	G	A	A	Y	syn
ATPase8	8414	C to T	C/A/C/A	C	T	T	Y	L-F
ATPase6	8701	A to G	A/I/F/T	A	G	G	Y	T-A
COIII	9824	T to A	T/F/L/C	T	A	A	Y	syn
ND3	10398	A to G	A/E/E/A	A	G	G	Y	T-A
	10400	C to T	C/I/T/E	C	T	T	Y	syn
ND4	10873	T to C	T/T/T/C	T	C	C	Y	syn
	11984	T to A	T/A/S/T	T	A	T	Y	Y-H
ND6	14580	A to T		A	T	T	Y	syn
	14587	A to C		A	C	C	Y	syn
	14605	A to G		A	G	G	Y	syn
	14668	C to T		C	T	T	Y	syn
Cytb	14783	T to C	T/F/K/A	T	C	C	Y	syn
	15043	G to A		G	A	A	Y	syn
	15301	G to A		G	A	A	Y	syn
	15326	A to G	A/L/S/A	A	G	G	Y	T-A
D-loop	16223	C to T		C	T	T	Y	
	16311	T to C		T	C	C	Y	
	16362	T to C		T	C	C	Y	

*Data show the conservation of amino acid for polypeptides or nucleotide for rRNAs, in human (H), mouse (M), bovine (B), and Xenopus laevis (X).

^†^Cambridge sequence.

^‡^See http://www.mitomap.org.

## Discussion

EH is currently regarded as a multifactorial CVD, which may be influenced by both genetic and/or environmental factors. The role of genetic factors in the etiology of hypertension is improved by cross-sectional studies of familial aggregation of the disorder despite exposure to different environmental factors [10,25]. Estimates of genetic variance range from 20–50% [26], and significant blood pressure correlations between mothers and offspring or excess maternal transmission of hypertension have been noted in several earlier [8,25] and more recent [8-12] investigations.

In this study, we performed the clinical, genetic molecular investigation of a Chinese family with hypertension. Hypertension as a sole clinical phenotype was only presented in all matrilineal relatives of this three-generation pedigree, suggesting that the mtDNA mutation (s) is involved in this disorder. Mutational analysis of the mitochondrial genome identified the novel mutation 4329C > G in tRNA^Ile^ and tRNA^Gln^ in matrilineal relatives of this Chinese family. The absence of this mutation from 366 Chinese controls strongly suggests that it is involved in the pathogenesis of hypertension. There are 32 variants identified belonging to the Eastern Asian haplogroup H2 on the maternal lineage. Thirty variants were the known polymorphisms, 4329C > G and 4467C > A were novel variants, but 4467C > A were not identified in other maternal members (not detected in II-8, II-13). The 4329C > G is located in the acceptor arm of tRNA^Ile^ and tRNA^Gln^, which is a 7-base pair stem made by the base pairing of the 5′-terminal nucleotide with the 3′-terminal nucleotide. This contains the CCA 3′-terminal group that is used to attach the amino acid. Pre-tRNA 3’ ends are subsequently completed by the addition of the CCA triplet, and a subset of nucleotides becomes post-transcriptionally modified. Finally, mature and correctly folded tRNAs are esterified at the 3’ end with the cognate amino acid by the corresponding aminoacyl-tRNA synthetase. These are subsequently transported to the ribosome by the translation factor elongation factor (EF)-Tu, enabling protein synthesis to occur. In addition, 4329 was located at the cleavage sites of tRNAase Z, so this substitution could impair (or not) the cleavage and processing of the policistronic mtRNA transcripts into mature mt-tRNA species. Thus, 3’ end metabolism is important for both tRNA synthesis and function, as shown by a previous report by Guan et al. in which a Chinese family with Leber’s hereditary optic neuropathy was found to be associated with the homoplasmic A15951G mutation located adjacent to the 3’ end [27].

## Conclusions

In summary, the occurrence of the 4329C > G mutation in the Chinese Han family pedigree with hypertension and its absence in 366 Chinese controls and the observed mitochondrial dysfunctions in cells carrying the mutation suggest that it is involved in the pathogenesis of hypertension. However, as hypertension is a complex CVD associated with multiple genetic and environmental factors, disease pathogenesis may result from this point mutation in association with other modifier factors.

## Abbreviations

EH: Essential hypertension; mtDNA: mitochondrial DNA; ECG: Electrocardiography.

## Competing interests

The authors declare that they have no competing interests.

## Authors’ contributions

JG, YLan carried out the molecular genetic studies, participated in the sequence alignment and drafted the manuscript. JY, ZL, CZ carried out the immunoassays. YLi and MG drafted the manuscript. YLi, MG and YC designed the whole experiments. All authors read and approved the final manuscript.

## Pre-publication history

The pre-publication history for this paper can be accessed here:

http://www.biomedcentral.com/1471-2350/15/84/prepub
